# Relationship between postprandial glucose level and carotid artery stiffness in patients without diabetes or cardiovascular disease

**DOI:** 10.1186/1471-2261-13-11

**Published:** 2013-02-26

**Authors:** Kentaro Watanabe, Tatsuya Suzuki, Motoshi Ouchi, Kazunari Suzuki, Makoto Ohara, Masao Hashimoto, Hidetoshi Yamashita, Masaru Okazaki, Kazuhito Ishii, Kenzo Oba

**Affiliations:** 1 Department of Internal Medicine (Divisions of Cardiology, Hepatology, Geriatrics, and Integrated Medicine), Nippon Medical School, 1-1-5 Sendagi, 113-8603, Bunkyo-ku, Tokyo, Japan

**Keywords:** 1,5-anhydroglucitol, Pulsatility index, Postprandial glucose, Nondiabetic patients

## Abstract

**Background:**

The aim of this study was to evaluate the relationship between postprandial glucose level and atherosclerosis in patients without diabetes and cardiovascular disease by determining carotid ultrasonographic variables and serum levels of 1,5-anhydroglucitol (1,5-AG).

**Methods:**

The subjects were 72 patients without diabetes and cardiovascular disease being treated for hypertension or dyslipidemia. The clinical characteristics of all subjects, including the serum level of 1,5-AG, which appears to be well suited for monitoring postprandial hyperglycemia, were evaluated after an overnight fast. The average intima-media thickness (IMT) and the average pulsatility index (PI) of the right and left common carotid arteries were determined with high-resolution ultrasonography and used as ultrasonographic variables. The subjects were divided into a Lower 1,5-AG group (n = 36) and a Higher 1,5-AG group (n = 36). We evaluated the relationship between clinical characteristics and ultrasonographic variables of the carotid artery in both groups.

**Results:**

The average PI in the Lower 1,5-AG group was significantly higher than that in the Higher 1,5-AG group, but the average IMT did not differ between the groups. Linear regression analysis, with the ultrasonographic variables as the dependent variables, with 1,5-AG as the independent variable, and adjusted for other clinical characteristics, showed significant correlation between 1,5-AG and the PI but not between 1,5-AG and IMT.

**Conclusion:**

Our results suggest that postprandial hyperglycemia increases carotid artery stiffness, but not morphological change, in patients without diabetes or cardiovascular disease.

## Background

Postprandial hyperglycemia is a major risk factor for morbidity and mortality due to cardiovascular disease in patients with diabetes [1,2]. Furthermore, in persons with impaired glucose tolerance (IGT), postprandial hyperglycemia, but not fasting hyperglycemia, increases the risk of cardiovascular disease [2]. Accordingly, decreasing postprandial hyperglycemia reduces the risk of cardiovascular disease in persons with either IGT [3] or diabetes [2]. We have recently found that brachial artery flow-mediated dilation is reduced after glucose loading and is negatively correlated with plasma glucose levels even in persons with normal glucose tolerance [4]. Hence, to reduce the risk of cardiovascular disease, the relationship between postprandial hyperglycemia and cardiovascular risk factors, such as atherosclerosis, should be evaluated. In fact, many tools are available for evaluating and predicting cardiovascular risk. For example, noninvasive ultrasonographic techniques can be used to evaluate the severity of vascular damage and to indicate the risk of progression of organ and vascular damage [5,6]. Carotid ultrasonography is a noninvasive and inexpensive examination and is widely available in outpatient clinics. The intima-media thickness (IMT) of the carotid artery is an ultrasonographic variable useful for evaluating vascular morphological changes and predicting cardiovascular disease [7]. In addition, the pulsatility index (PI) of the carotid artery is a hemodynamic variable that is easily measured with Doppler ultrasonography and is considered to reflect peripheral aortic stiffness distal to the measurement point [8]. Hence, the IMT and PI of the carotid artery are suitable variables for morphological and functional assessment of carotid atherosclerosis.

The aim of the present study was to evaluate the relationship between postprandial glucose levels and atherosclerosis in patients without diabetes or cardiovascular disease by determining the IMT and PI of the common carotid artery (CCA).

## Methods

### Study subjects

The subjects were 72 patients (24 men and 48 women; average age, 69.4 ± 10.7 years) without diabetes or cardiovascular disease being treated for hypertension or dyslipidemia at the outpatient clinic of our division. All subjects were ambulatory and were free of anorexia or stress conditions possibly affecting glycemic conditions. All subjects were considered not to have diabetes because they met none of the following criteria in the past: 1) fasting plasma glucose level ≥ 7.0 mmol/L; 2) 2-hour value ≥ 11.1 mmol/L on the 75-g oral glucose tolerance test; 3) casual plasma glucose level of ≥ 11.1 mmol/L; and 4) A1C (NGSP) ≥ 6.5%. The glycemic conditions of all subjects were stable from the time of a non-diabetes diagnosis to the start of the study period.

[9]. Furthermore, subjects were excluded on the basis of the following criteria: pregnancy, previous gastrectomy, anemia, severe illness, serum creatinine ≥ 114.92 μmol/L, urine protein test > 1+ (equivalent to > 0.3 g/L), renal glucosuria, liver cirrhosis, chronic hepatitis, and the use of drugs, such as oral hypoglycemic agents, steroids, and traditional Chinese herbal medicines.

### Study design

#### Informed consent and ethics regulations

The study design was approved by the Ethics Committee of Nippon Medical School and carried out in accordance with the principles of the Declaration of Helsinki. Before the start of the study, written informed consent was obtained from all subjects after they had received a clear explanation of the study protocol.

#### Measurement of carotid ultrasonographic variables

The IMT and PI of CCA were determined as carotid ultrasonographic variables as previously reported [10]. Carotid ultrasonographic measurements were performed with a high-resolution ultrasonographic scanner and a linear-array 8-MHz transducer (SSA-350A, Toshiba Medical Systems, Co., Ltd, Tokyo, Japan). The IMT was measured at a total of 4 segments clearly visualized with B-mode imaging of the near and far walls immediately proximal to the carotid bifurcation in the right and left CCAs. The average of the 4 IMT measurements was calculated and defined as the IMT [11].

The PI of the CCA was determined with pulse Doppler ultrasonography. Pulse Doppler volume measurements were performed with a maximum angle of less than 60 degrees at the same points where the IMT had been measured. The sample-volume measurement point was placed at the center of CCA flow, and peak systolic flow velocity (PSV), end-diastolic flow velocity (EDV), and time-averaged flow velocity (TAV) were determined on the basis of sample volumes. The PI was calculated as follows: PI = (PSV-EDV)/TAV. The average of values from the right and left CCAs was calculated and defined as the PI [10].

Carotid ultrasonographic measurements were performed by 2 observers. The correlation coefficient for interobserver reproducibility and the variability of measurements, respectively, were r = 0.896 (P < 0.001) and 8.0% for the IMT and r = 0.979 (P < 0.001) and 5.8% for the PI.

#### Measurement of pulse wave velocity

To evaluate the relationship between PI and vascular stiffness, the pulse wave velocity (PWV) of the study subjects was evaluated with an automated device (form PWV/ABI; Omron Colin Co., Ltd., Tokyo, Japan) immediately after the carotid ultrasonographic variables were measured, as previously reported [12]. We measured and evaluated the average right and left brachial-ankle PWVs (baPWVs) of 58 of 72 study subjects.

#### Evaluation and classification of postprandial glucose levels

Postprandial glucose levels were determined on the basis of 1,5-anhydroglucitol (1,5-AG), a major human polyol. From 99% to 100% of 1,5-AG is reabsorbed in normoglycemia, but the reabsorption rate decreases significantly in hyperglycemia in approximate proportion to the degree of hyperglycemia above the renal threshold for glucosuria [13,14]. Therefore, the serum level of 1,5-AG appears to be well suited for monitoring glucose homeostasis in patients with near-normoglycemia [15,16] or postprandial hyperglycemia without fasting hyperglycemia [17,18]. Furthermore, serum 1,5-AG level has been suggested to be realted to microvascular and macrovascular complications [14]. Subjects with conditions, other than postprandial hyperglycemia, that effect serum 1,5-AG level had already been excluded on the basis of exclusion criteria. The serum 1,5-AG level was evaluated as previously reported [19]: after an overnight fast the 1,5-AG level was measured with an enzymatic method, (Lana 1,5-AG Auto Liquid, Nippon Kayaku, Tokyo, Japan) with an automatic clinical analyzer (model 7150, Hitachi High-Technologies Corporation, Tokyo, Japan). The overnight fasting period was defined as the 12 hours after the start of the last meal, as previosly reported [20]. On the basis of serum 1,5-AG levels, the subjects were divided into 2 groups: subjects with lower serum 1,5-AG levels (Lower 1,5-AG group; n = 36), and subjects with higher serum 1,5-AG levels (Higher 1,5-AG group; n = 36).

#### Clinical characteristics of study subjects

The clinical characteristics evaluated were age, sex, body-mass index (BMI), smoking habit, hypertension, statin use, systolic and diastolic blood pressures, and biochemical variables, including 1,5-AG and HbA1c (Japan Diabetes Society [JDS]). Biochemical variables were evaluated after an overnight fast. Serum total cholesterol, high-density lipoprotein (HDL) cholesterol, triglycerides, uric acid, and creatinine were measured with an automatic analyzer. The HbA1C (JDS) was measured with high-performance liquid chromatography (JDS Lot 3). Also, the HbA1c (JDS) was transformed into A1C (NGSP) as follows: A1C (NGSP) = HbA1c (JDS) + 0.4 [21].

### Statistical analysis

The Mann-Whitney U-test was used to compare clinical characteristics and ultrasonographic variables between the Lower and Higher 1,5-AG groups. Multivariate linear regression analysis was used to identify associations between carotid ultrasonographic variables and serum 1,5-AG levels. In this multivariate linear regression analysis, we assumed that ultrasonographic variables were dependent variables and that clinical characteristics, including the 1,5-AG level, were independent variables. Data are presented as means ± SD or β coefficients (95% confidence interval, CI). Statistical significance was defined as P < 0.05. All analyses were performed with SPSS for Windows Ver. 12.0 J (IBM SPSS Statistics, IBM Corp., Armonk, NY).

## Results

The average serum 1,5-AG level in the Lower 1,5-AG group (12.71 ± 3.54 μg/mL) was significantly lower than that in the Higher 1,5-AG group (23.16 ± 3.82 μg/mL, P < 0.001, Table 1). However, no other clinical characteristics differed significantly between the Lower and the Higher 1,5-AG groups.

**Table 1:** Clinical characteristics of study subjects

** Clinical characteristics**	**Lower 1,5-AG group (n = 36)**	**Higher 1,5-AG group (n = 36)**
Male sex	13 (36.1)	11 (30.6)
Age (years)	70.9 ± 10.2	67.9 ± 11.2
BMI (kg/m^2^)	23.8 ± 4.0	24.0 ± 5.1
Smoking habit		
None	20 (55.9)	25 (69.4)
Recent	10 (27.8)	6 (16.7)
Current	6 (16.7)	5 (13.9)
Hypertension	28 (77.8)	23 (63.9)
Statin use	16 (44.4)	14 (38.9)
Blood pressure (mm Hg)		
Systolic	134.3 ± 16.1	131.0 ± 12.6
Diastolic	79.9 ± 7.2	81.8 ± 7.4
Total cholesterol (mmol/L)	5.42 ± 0.59	5.64 ± 1.14
HDL cholesterol (mmol/L)	1.46 ± 0.43	1.60 ± 0.43
Triglycerides (mmol/L)	1.58 ± 0.68	1.46 ± 0.85
Uric acid (μmol/L)	317.6 ± 98.2	342.4 ± 82.4
Serum creatinine (μmol/L)	71.0 ± 15.7	76.4 ± 15.6
FPG (mmol/L)	5.16 ± 0.40	5.15 ± 0.43
A1C (%)	5.66 ± 0.32	5.57 ± 0.28
1,5-AG (μg/mL)***	12.71 ± 3.54	23.16 ± 3.82

Mean ± SD, n (%).

BMI: body-mass index; FPG: fasting plasma glucose; A1C: glycosylated hemoglobin; 1,5-AG: 1,5-anhydroglucitol.

***P < 0.001.

**Figure 1:**
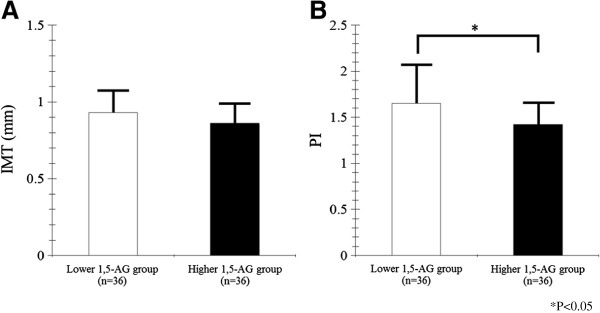
**The IMT and PI of subjects in the Lower and Higher 1,5-AG groups. A**: Comparison of IMT between the Lower and Higher 1,5-AG groups. **B**: Comparison of PI between the Lower and Higher 1,5-AG groups.

The average IMT in the Lower 1,5-AG group (0.92 ± 0.17 mm) was higher, but not significantly so, than that in the Higher 1,5-AG group (0.86 ± 0.14 mm; P = 0.066, Figure 1A). On the other hand, the average PI in the Lower 1,5-AG group (1.65 ± 0.43) was significantly higher than that in the Higher 1,5-AG group (1.42 ± 0.25, P = 0.013, Figure 1B).

Linear regression analysis adjusted for the subjects’ clinical characteristics showed significant correlations between 1,5-AG and PI (β = −0.017, t = −2.583; P = 0.012, Table 2) but not between 1,5-AG and IMT (Table 3).

**Table 2:** Multivariate linear regression analysis assuming PI as the dependent variable and 1,5-AG as the independent variable

**Pulsatility index**	**β Coefficient (95% confidence interval)**	**t-value**
Model 1**	−0.023 (−0.035 - -0.010)	−3.639
Model 2**	−0.018 (−0.030 - -0.006)	−3.023
Model 3*	−0.016 (−0.029 - -0.004)	−2.637
Model 4*	−0.017 (−0.029 - -0.004)	−2.631
Model 5*	−0.017 (−0.029 - -0.004)	−2.619
Model 6*	−0.018 (−0.031 - -0.004)	−2.607
Model 7*	−0.017 (−0.031 - -0.004)	−2.583

Model 1, unadjusted; Model 2, adjusted for age and sex; Model 3, adjusted for Model 2, BMI, and smoking habit; Model 4, adjusted for Model 3 and systolic BP; Model 5, adjusted for Model 4, statin use, and total cholesterol; Model 6, adjusted for Model 5 and serum creatinine; Model 7, adjusted for Model 6 and A1C.

*P < 0.05, **P < 0.01.

**Table 3:** Multivariate linear regression analysis assuming IMT as the dependent variable and 1,5-AG as the independent variable

**Intima-media thickness**	**β Coefficient (95% confidence interval)**	**t-value**
Model 1	−0.005 (−0.011 - 0.001)	−1.749
Model 2	−0.002 (−0.007 - 0.003)	−0.683
Model 3	−0.002 (−0.008 - 0.003)	−0.810
Model 4	−0.002 (−0.007 - 0.003)	−0.705
Model 5	−0.002 (−0.007 - 0.003)	−0.785
Model 6	−0.001 (−0.006 - 0.005)	−0.304
Model 7	−0.001 (−0.006 - 0.005)	−0.287

Model 1, unadjusted; Model 2, adjusted for age and sex; Model 3, adjusted for Model 2, BMI, and smoking habit; Model 4, adjusted for Model 3 and systolic BP; Model 5, adjusted for Model 4, statin use, and total cholesterol; Model 6, adjusted for Model 5 and serum creatinine; Model7, adjusted for Model 6 and A1C.

**Figure 2:**
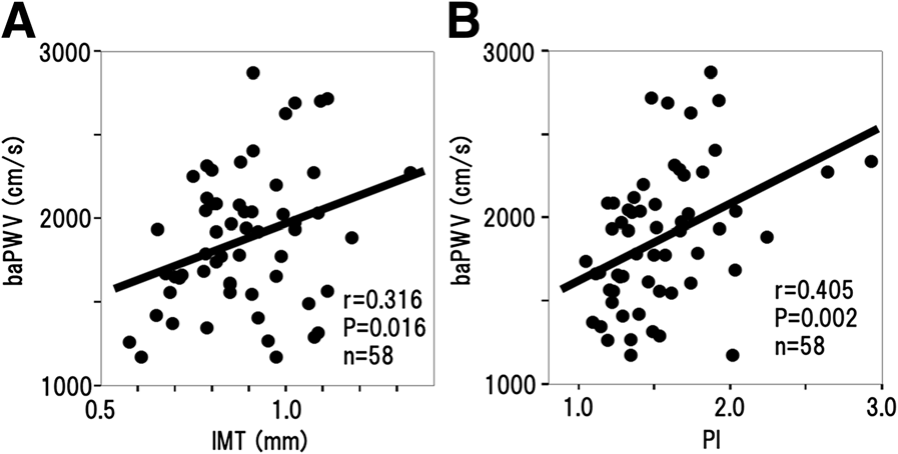
**Correlations between baPWV and carotid ultrasonographic variables. A**: Correlation between IMT and baPWV. **B**: Correlation between PI and baPWV.

Concerning baPWV in 58 study subjects, the average baPWV in the Lower 1,5-AG group (1929.0 ± 489.9 cm/s, n = 28) was higher, but not significantly so, than that in Higher 1,5-AG group (1812.6 ± 328.2 cm/s, n = 30; P = 0.673). However, PI was significantly correlated with baPWV (r = 0.405; P = 0.002, Figure 2B). In contrast, IMT was significantly, but weakly correlated with baPWV (r = 0.305; P = 0.016, Figure 2A).

## Discussion

Our results demonstrate that the serum 1,5-AG level in persons without diabetes or cardiovascular disease is significantly correlated with the PI of the carotid artery but not with the IMT.

Increased vascular stiffness is an early change in atherosclerosis [6]. In particular, several studies have shown that the PI of the carotid artery is significantly correlated with cerebrovascular disease [10,22,23]. Furthermore, the PI of the carotid artery was significantly correlated with Framingham risk scores in subjects with hypertension [24], and systemic artery stiffness is correlated with carotid artery stiffness [25], cardiovascular risk score, and the presence of cardiovascular disease [26]. The present study also found that the PI was significantly correlated with the baPWW. Furthermore, aortic stiffness is an indicator of cardiovascular disease, and high aortic stiffness is correlated with an increased risk of cardiovascular events [27]. On the other hand, reduced stiffness of the CCA is associated with a reduced risk of cardiovascular events [28]. Hence, the relation between carotid artery stiffness and cardiovascular disease suggests that the increased PI of the carotid artery in the present subjects is associated with an increased risk of cardiovascular disease. Our results in subjects without diabetes or cardiovascular disease also suggest that the risk of cardiovascular disease is positively correlated with vascular stiffness, which is induced by postprandial hyperglycemia, rather than with the morphological changes of atherosclerosis. In fact, Li et al. have found that the baPWV is increased in subjects with IGT or newly diagnosed diabetes but not in subjects with normal glucose tolerance or isolated impaired fasting glucose [29]. Furthermore, Huang et al. have found that the vascular stiffness of healthy individuals is significantly and positively correlated with glucose levels 60 minutes after oral glucose challenge [30].

The mechanism of increased vascular stiffness in the absence of the morphological changes of atherosclerosis in the present study remains unclear. However, several previous studies have provided important information regarding the mechanism of how postprandial hyperglycemia increases vascular stiffness. Endothelium-derived nitric oxide (NO) is considered an important factor in the relaxation of vascular smooth muscle cells. The proposed mechanism of relaxation is that NO induces the hyperpolarization of smooth muscle cells by reducing the open probability of Ca-channel–dependent activation of the sarcoplasmatic reticulum which, in turn, decreases Ca^2+^ influx [31]. Many studies have found that acute hyperglycemia is associated with increased oxidative stress, which inactivates NO and contributes to endothelial cell injury *in vitro*[32,33]. In particular, Ceriello et al. have found that glucose levels fluctuating over 24 hours are more deleterious to endothelial function and oxidative stress than are continuously high glucose concentrations in healthy persons or patients with type 2 diabetes [34]. Azuma et al. have shown that repeated fluctuations in glucose or insulin increase monocyte adhesion to the endothelium of the rat thoracic aorta and that stable hyperglycemia or hyperinsulinemia causes less monocyte adhesion [35]. Furthermore, Ge et al. have reported that oxidative stress under intermittently high glucose conditions is significantly greater than that under constantly high glucose conditions *in vitro*[36]. Such differences have also been found in healthy subjects with normal glucose tolerance [37-39] and in persons with IGT [38,39] or diabetes [40,41]. In fact, we have previously demonstrated that oral glucose loading attenuates brachial artery flow-mediated dilation in persons with normal glucose tolerance [4]. Our present findings in subjects without the morphological changes of atherosclerosis suggest that impaired NO bioactivity due to acute glucose elevation in the postprandial state increases carotid vascular stiffness.

The present study had several limitations. First, we could not assess the associations of OGTT plasma glucose levels, with vascular stiffness. This relationship may provide additional information against the results of the present study. Second, we could not assess the relationship between lipid metabolism and carotid artery stiffness. Third, the final meal on the evening before the study was not standardized. 1,5-AG in the body originates mainly from foods and is well absorbed in the intestine. The daily intake of 1,5-AG is approximately 26.8 μmol and is independent of food type (13.4 μmol/100 kcal) [42]. On the other hand, the serum 1,5-AG level is correlated with the daily urinary glucose excretion level [16]. Therefore, a difference in energy intake or the glycemic index might produce a difference in daily urinary glucose excretion. Finally, the number of subjects in our study was small; therefore, the results of our study should be clarified in a larger population.

## Conclusion

The results of the present study suggest that postprandial hyperglycemia increases carotid vascular stiffness in patients without diabetes or cardiovascular disease. Further prospective study is needed to confirm these results.

## Abbreviations

IMT: Intima-media thickness;PI: Pulsatility index;IGT: Impaired glucose tolerance;CCA: Common carotid artery;baPWV: Brachial-artery pulse wave velocity;1,5-AG: 1,5-anhydroglucitol;NO: Nitric oxide

## Competing interests

The authors declare that they have no conflict of interest for this study.

## Authors’ contributions

KW edited this manuscript. TS and KO contributed to the study conception and design. MO contributed to the study conception and design. KS, MO, and MH participated in data collection, data analysis and interpretation, and drafted the manuscript. HY participated in data collection, data interpretation, and edited the manuscript. MO and KI participated in data correction. All authors read and approved the final manuscript.

## Pre-publication history

The pre-publication history for this paper can be accessed here:

http://www.biomedcentral.com/1471-2261/13/11/prepub
